# Bedside detection and monitoring of pulmonary embolism using electrical impedance tomography

**DOI:** 10.3389/fphys.2025.1729553

**Published:** 2026-01-28

**Authors:** Mingyuan Deng, Nianze Li, Jiafeng Wang, Shuang Zhao, Mingjing Yu

**Affiliations:** 1 West China School of Medicine/West China Hospital, Sichuan University, Chengdu, China; 2 Department of Respiratory and Critical Care Medicine, Institute of Respiratory Health, State Key Laboratory of Respiratory Health and Multimorbidity, West China Hospital, Sichuan University, Chengdu, China

**Keywords:** bedside monitoring, critical care, electrical impedance tomography (EIT), pulmonary embolism (PE), pulmonary perfusion

## Abstract

Pulmonary embolism (PE) is a common and potentially fatal obstructive disease of the pulmonary arteries; early diagnosis and continuous monitoring are particularly critical in critically ill patients. Electrical impedance tomography (EIT), a noninvasive and radiation-free imaging modality that enables real-time bedside monitoring, offers a promising approach for adjunctive diagnosis and perfusion assessment of PE, especially in patients who cannot undergo computed tomography pulmonary angiography (CTPA) due to instability or other contraindications. Building upon an overview of EIT imaging principles and recent advances in pulmonary perfusion monitoring, this review concentrates on the bedside application of EIT and the clinical value of EIT in bedside assessment of PE. Unlike prior research, this study proposes an EIT perfusion imaging strategy using a hypertonic saline bolus for the diagnosis of PE and compares it with bedside monitoring based on cardiac impedance signals. Additionally, we assess the current clinical evidence according to GRADE standards and identify its existing limitations. Finally, we further discuss the key challenges hindering clinical translation of EIT and outline future directions. This review aims to provide clinicians and researchers with a reference to facilitate broader adoption of EIT in the bedside monitoring of PE.

## Introduction

Pulmonary embolism (PE) is a common and highly fatal acute pulmonary vascular disease, particularly prevalent among critically ill and high-risk patients. Early diagnosis and timely intervention are crucial for improving outcomes. At present, the clinical diagnosis of PE primarily relies on computed tomography pulmonary angiography (CTPA) and ventilation/perfusion (V/Q) scintigraphy. However, such examinations are often restricted by the availability of imaging equipment, the risk of patient transfer, and the use of contrast agents, making it difficult to be widely applied among critically ill patients.

Electrical impedance tomography (EIT) is an emerging, noninvasive, radiation-free clinical tool to image, in real time and at the bedside. In recent years, it has increasingly drawn attention in critical care medicine, respiratory monitoring, and lung function assessment. In addition to its common role in pulmonary ventilation monitoring, researchers have gradually applied EIT to pulmonary perfusion imaging, offering a new approach to evaluating pulmonary blood flow. Preliminary studies and case reports suggest that EIT holds potential value for bedside identification and dynamic monitoring of PE.

To clarify the clinical value and application pathways of EIT in bedside monitoring of PE, this review follows the analytical framework. First, based on a description of the technical principles, we systematically review the research progress of EIT in pulmonary perfusion monitoring, with a focus on comparing the strengths, limitations, and applicable scenarios of the two technical approaches that can evaluate the specific changes in lung perfusion. Second, we synthesize current clinical evidence on the use of EIT to identify perfusion defects and quantify the extent of regional ventilation–perfusion (V/Q) mismatch in patients with suspected PE, while outlining the diagnostic parameters employed and their inherent limitations. Finally, drawing on existing research consensus, we discuss the feasible pathways and future directions for the clinical translation of EIT.

## Overview of electrical impedance tomography

EIT is an imaging modality that reconstructs the distribution of tissue impedance in the body based on impedance changes of biological tissues under different physiological and pathological conditions. Its fundamental principle relies on differences in electrical conductivity and material properties to generate image contrast (Cui et al., 2024). In biomedical applications, EIT essentially reflects the dynamic distribution of tissue impedance, which is closely related to electrophysiological properties. Pathological changes in tissue composition, such as pleural effusion, pulmonary fibrosis, or pulmonary edema, may cause significant local impedance alterations (Lobo et al., 2018). On account of these properties, EIT systems can apply safe, low-intensity alternating currents to human tissues, record the resulting voltage distributions caused by the stimulating current, and reconstruct images according to the obtained data. This approach enables noninvasive, dynamic monitoring of physiological and pathological states in human tissues and organs (Bachmann et al., 2018).

Due to its real-time and bedside operable features, EIT has rapidly evolved in the diagnosis and management of lung diseases, cardiovascular and cerebrovascular diseases, and other fields, serving as a valuable complement to conventional imaging methods. Currently, one of its most established applications is ventilation/perfusion (V/Q) monitoring (Cui et al., 2024). In pulmonary medicine, EIT-based ventilation monitoring is widely used for the management of critical care respiratory conditions. More recently, its clinical utility has expanded to include pulmonary perfusion assessment and cardiopulmonary interaction monitoring.

In bedside monitoring settings, EIT continuously acquires data through a surface electrode array and reconstructs two-dimensional dynamic images that reflect regional changes in tissue impedance. These images intuitively depict the distribution of ventilation or perfusion across different lung regions and are commonly used to assess lung asymmetry, perfusion defects, and related abnormalities. Precisely, its application in PE is based on its potential for identifying uneven perfusion.

## Research on the application of EIT in pulmonary perfusion monitoring

EIT was initially applied mainly for monitoring pulmonary ventilation. With advances in reconstruction algorithms and gating techniques, its application in pulmonary perfusion assessment has gradually attracted increasing attention. EIT can provide bedside, noninvasive, and continuous images related to lung perfusion, providing a supplement to conventional imaging methods such as CTPA, magnetic resonance imaging (MRI), V/Q scintigraphy, and is particularly suitable for dynamic monitoring of critically ill patients.

According to current expert consensus, EIT-based lung perfusion assessment refers specifically to methods involving intravenous bolus injection of impedance contrast agents (He et al., 2025). Another technique utilizes cardiac pulsation signals for bedside dynamic monitoring of relative perfusion trends. The following section will systematically compare these two approaches (Table 1).

**TABLE 1:** Comparison of EIT-based technical pathways for pulmonary blood flow assessment and monitoring.

Approaches for pulmonary blood flow assessment and monitoring	Contrast-enhanced electrical impedance tomography method	Cardiac pulsation-related signal-based method
Principle	Injecting hypertonic saline bolus through a central venous catheter As the contrast agent first passes through the pulmonary circulation, it produces a characteristic transient drop in thoracic impedance, reflecting regional perfusion status (Xu et al., 2021)	Specific algorithms extract the impedance variation synchronized with cardiac pulsation from mixed EIT signals. This component reflects periodic fluctuations in pulmonary blood volume and is used to monitor relative changes in regional lung perfusion. (She et al., 2023)
Pulmonary perfusion impedance operation requirements	Usually, 10 mL of hypertonic (e.g., 5%–10%) normal saline is used as the contrast agent (He et al., 2020a) (Wang et al., 2022) Central venous access is required for the injection of hypertonic saline (He et al., 2020b) The contrast agent should be injected in a bolus within at least 8 s of breath-holding to obtain EIT images (He H. W. et al., 2021) (He H. et al., 2021)	Deploy the surface EIT electrode array and the electrocardiogram gating system The data collection and analysis methods include respiratory pause (RP), electrocardiogram-gated EIT (ECG-gated EIT), frequency-domain filtering (FDF) and Principal component analysis (PCA) (He H. W. et al., 2021) (McArdle et al., 1988; Zadehkoochak et al., 1992; Jang et al., 2020)
Clinical applicable population	Patients undergoing mechanical ventilation with intubation and sedation (He et al., 2020b)	Applicable to both conscious patients and those on mechanical ventilation
Function and effect evaluation	A prospective observational clinical study has confirmed that the EIT hypertonic saline contrast method has a high diagnostic efficacy for PE (He et al., 2020b)	Animal model experiments have verified the feasibility and accuracy of the periodic pulmonary vascular pulsation method for monitoring the periodic pulmonary blood volume fluctuations (Fagerberg et al., 2009)
Signal-to-noise ratio (SNR)	High (Muders et al., 2023)	Low to medium (She et al., 2023)
Advantage	Can directly track the forward pulmonary blood flow rather than merely measuring the pulsation changes of pulmonary blood volume More sensitive to regional perfusion abnormalities	Non-invasive Can monitor the specific changes in lung perfusion in real time
Main limitations	It may be difficult for the patient to maintain respiratory pauses during the process of spontaneous breathing (Xu et al., 2021; He H. et al., 2021)	Not sensitive to peripheral subsegmental PE; Susceptible to the expansion effect of pulmonary vessels; Data processing is relatively complex (She et al., 2023). Not for independent diagnosis

At present, both experimental studies and clinical investigations are ongoing worldwide to further explore EIT-based pulmonary perfusion assessment, aiming to verify its feasibility and clinical utility.

### Normal human and animal experimental research

Early animal studies laid essential groundwork for the use of EIT in pulmonary perfusion assessment, providing preliminary validation of its feasibility. Research conducted in porcine and ovine models demonstrated that EIT can reliably detect abnormalities in regional perfusion distribution, with good concordance between EIT-derived measurements and reference imaging modalities such as SPECT and PET (Frerichs et al., 2002; Stowe et al., 2019; Borges et al., 1985; Bluth et al., 2019). Subsequent investigations have progressively advanced toward optimizing imaging methodologies and contrast agent selection. These animal experiments not only facilitated the evolution of EIT from two-dimensional to three-dimensional imaging (Larrabee et al., 2023) but also explored safer contrast alternatives such as sodium bicarbonate (NaHCO_3_) (Gao et al., 2025), establishing an important foundation for future clinical translation of this technology.

### Perfusion assessment in clinical scenarios

In the intensive care unit (ICU), the real-time dynamic monitoring capability of EIT is of particular importance. By integrating ECG gating technology, EIT can provide perfusion waveforms in multiple lung regions and relative V/Q matching status, offering an important basis for clinical assessment of V/Q mismatch. In a prospective study, researchers evaluated the reliability of ECG-gated EIT in measuring stroke volume in critically ill patients. The results showed good agreement with measurements obtained by transpulmonary thermodilution (TPTD), supporting the feasibility of using regional impedance changes synchronized with cardiac activity to estimate stroke volume variations (Braun et al., 2020). Marco Leali and colleagues further proposed a noninvasive ECG-gated EIT calibration method that integrates ECG signals with EIT data to generate absolute V/Q images (V/Q-abs). The calibrated V/Q-abs images closely approximated those obtained from invasive monitoring, demonstrating the potential of this approach to achieve completely noninvasive quantification of V/Q matching for dynamic bedside perfusion monitoring (Leali et al., 2024).

### Technical parameters and imaging methods

Acquisition of pulmonary perfusion images with EIT typically requires the integration of several technical methods:

Respiratory pause (RP): Images are obtained during brief apnea to minimize respiratory interference and improve perfusion signal contrast (He H. W. et al., 2021).

Contrast enhancement: Injection of 15–20 mL of cold saline as a conductive contrast agent temporarily alters impedance distribution, enhancing the contrast between perfused regions and surrounding tissues (He H. W. et al., 2021).

Electrocardiogram-gated EIT (ECG-gated EIT): By synchronously recording ECG signals, impedance changes across multiple cardiac cycles can be averaged to improve image stability (McArdle et al., 1988).

Frequency-domain filtering (FDF): Since ventilation and perfusion signals differ in frequency, filters are applied to separate these components in the frequency domain from the raw data (Zadehkoochak et al., 1992).

Principal component analysis (PCA): Variance-based analysis of EIT time-series data separates respiration-related impedance changes (e.g., tidal volume variations) from cardiac-related changes (e.g., pulmonary vascular filling/emptying) into distinct principal components (Jang et al., 2020).

Currently, most EIT devices employ 16- or 32-electrode systems, which limit image resolution. Nevertheless, their advantages lie in bedside applicability and the ability to perform serial measurements at multiple time points, making them particularly suitable for dynamically tracking changes in pulmonary perfusion.

### The empowerment of artificial intelligence and targeted analysis

In recent years, with the continuous breakthroughs in deep learning algorithms, the integration of AI with EIT has been constantly developing, and its application scope in medical imaging and the treatment of pulmonary diseases has gradually expanded. Researchers have proposed an enhanced EIT approach known as single-network reconstruction, in which a neural network directly reconstructs images from raw EIT data. By learning the complex relationship between surface electrical measurements and internal conductivity distributions, the network is able to generate high-resolution images that are critical for diagnostic purposes (Zheng et al., 2022).

AI also shows considerable potential in pulmonary feature extraction. In the future, AI-based algorithms may process EIT data to extract clinically relevant parameters such as global inhomogeneity (GI), center of ventilation (CoV), regional ventilation delay (RVD), tidal impedance variation (TIV), and end-expiratory lung impedance (EELI). These parameters are essential for assessing lung function and may contribute to optimizing patient management and improving clinical outcomes (Cappellini et al., 2024). Furthermore, these AI-enabled quantitative features may be integrated with the characteristic pattern of focal perfusion defects seen in PE, thereby enabling automated detection and risk alerting on top of AI-assisted interpretation. Such integration is expected to enhance the capability of EIT for early bedside identification of PE and for dynamic risk assessment throughout the clinical course.

## The initial exploration of EIT in bedside monitoring on pulmonary embolism

The most common cause of PE is deep vein thrombosis (DVT), in which thrombi originating from the deep veins of the lower limbs or pelvis detach and obstruct the pulmonary arteries via the circulation (Walter, 2023). This further leads to an increase in pulmonary artery pressure, damaging the structure and function of the right heart, which can even cause right heart failure and death. Improving the diagnosis and management of PE is critical to reducing PE-related mortality and recurrence, improving patient outcomes, and alleviating the healthcare burden (Konstantinides et al., 2020). In clinical practice, the diagnosis of PE relies heavily on imaging modalities such as CTPA or V/Q scans. However, such examinations often require the transfer of patients or the use of contrast agents, which may pose certain risks, especially in critically ill or bedridden patients. EIT, as a novel bedside, noninvasive, and dynamic monitoring technique, provides a promising alternative for adjunctive diagnosis and perfusion assessment of PE.

### Overview of case studies and small sample research

In this review, we define studies with a sample size <20, whether prospective or retrospective, as small clinical studies to differentiate them from single-case reports. For clarity and transparency, the sample size of each study has been explicitly indicated in the table (Table 2).

**TABLE 2:** Case reports and small clinical studies of EIT applied in PE.

Author (Year)	Sample capacity	Patient characteristics	Reference diagnosis	EIT method	Key EIT findings (V/Q status)	EIT timing and role	Clinical course	Prognosis
Safaee et al. (2020)	1	66-year-old male, ICU intubated, COVID-19; EIT:homogeneous ventilation, dead space 66%, RUL V/Q mismatch	Echocardiography: elevated RVSP, PH; CTPA (day 8): emboli in RUL branches	Hypertonic saline (10 mL, 10%)	Uneven perfusion: left 64%, right 36%	The first EIT was performed on the day of clinical deterioration (2 days prior to CTPA confirmation), followed by a second examination on the day of CTPA diagnosis	UFH; EIT (day 17) improved; CTPA (day 34) resolved	Discharged (day 68)
He et al. (2020c)	1	47-year-old female, POD1 after LUL lobectomy; dyspnea, hypoxemia	CTPA: multiple emboli in right PA branches	Hypertonic saline (10 mL, 10%)	Right lung V/Q mismatch	The EIT examination was conducted 4 h after the CTPA diagnosis		
He H. et al. (2021)	11	Prospective study, 11 intubated ICU patients	ten confirmed PE by CTPA, one by history + ultrasound	Hypertonic saline (10 mL, 10%)	EIT: PE group higher dead space %, lower shunt %, lower V/Q match % vs. non-PE			
Grassi et al. (2020)	1	57-year-old female, intraop left nephrectomy, hemodynamic instability	CTPA: embolus in LPA	Small saline bolus	Perfusion defect in LUL; V/Q 22%/7%	EIT as supplementary assessment post-CTPA.		
Foronda et al. (2022)	1	15-year-old female, COVID-19, hypoxemia (day 4), asymmetric breath sounds	CTPA: filling defect LPA	Cardiac pulsation-related signal-based method (ECG-gated averaging)	Left perfusion defect	EIT as supplementary assessment post-CTPA.	LMWH	Improved, transferred to ward (day 4)
Yuan et al. (2021)	1	68-year-old male, POD1 after cancer surgery, cardiac arrest	Echo: RV enlargement, fixed IVC; CTPA: bilateral main PA emboli	Hypertonic saline (10 mL, 10%)	Bilateral V/Q mismatch, perfusion improved after anticoag	EIT performed immediately upon ICU admission	UFH	Discharged (day 25)
Wang et al. (2021)	1	64-year-old male, bladder/prostate cancer, POD6, Wells 7, sudden hypoxemia	CTPA: thrombi in right main PA and bilateral branches	Hypertonic saline (10 mL, 10%)	Pre-thrombolysis: right defect, dead space 28.8%; improved post-thrombolysis	EIT performed on day of deterioration, prior to CTPA.	Alteplase + UFH	CTPA improved; transferred to ward (day 15)
Kuk and Wright (2022)	1	42-year-old male, POD3 intubated, hypoxemia during vent optimization	Echo: mild RV dilation, PH; CTPA (day 8): multiple segmental emboli	Hypertonic saline (10 mL, 7%)	Pre-treatment right anterior V/Q 21%/1%	EIT (POD4) detected V/Q mismatch, guided diagnosis before CTPA (POD8)	Heparin	ICU discharge (day 15), hospital discharge (day 17)
Manuel et al. (2024)	1	44-year-old male, CTEPH after PTE, ICU respiratory failure	Echo: normal biventricular; CXR: normal	Hypertonic saline (10 mL, 7.5%)	Baseline: L V/Q 54%/28%, R V/Q 46%; after PEEP ↑10 mmHg: distribution improved, balanced V/Q	EIT with the ionic contrast method was performed 1 h postoperatively	PEEP + anticoag	Rehab after ICU discharge
Prins et al. (2023)	1	64-year-old male, COVID-19 + OSA, ICU intubated	Echo: RV dilatation; unstable, no CTPA	Hypertonic saline (10 mL, 7.5%)	R lung V/Q 65%/22% pre-lysis; post-lysis 70%/43%	EIT guided bedside ventilation optimization. CTPA was deferred due to hemodynamic instability	Thrombolysis; oxygenation improved	Died (day 19) from comorbidities
Ding et al. (2024)	1	72-year-old male, ICU intubated, colon cancer history, shock; Geneva score 10	Echo: mild RV dilation, IJ thrombosis; renal failure, no CTPA	Hypertonic saline (10 mL, 10%)	Pre-anticoag: R dead space 14.7%; EIT: persistent hypoperfusion		LMWH→UFH; US resolution (day 2); CTPA (day 28): subsegmental emboli	Transitioned to OAC after discharge
Wang et al. (2024)	1	25-year-old female, CTEPH, WHO IV, post-PEA with *in-situ* thrombosis	CTPA: RPA occlusion	Hypertonic saline (10 mL, 10%)	R perfusion defect, V/Q mismatch			Family declined further surgery
Magaña et al. (2025)	1	76-year-old male, syncope, ER presentation	CT: bilateral PE, worse on R		R upper lobe perfusion defect (V/Q mismatch)		Anticoag + PEEP	Perfusion normalized
Garberi et al. (2025)	1	54-year-old male, severe hypoxemia on VV-ECMO; R pneumonia + L PE	CT: R pneumonia, CECT: LPA thrombus	Hypertonic saline (10 mL, 5%)	V/Q mismatch: ventilation L, perfusion R		EIT (day 2): mismatch persisted; after thrombectomy, V/Q normalized	Transferred to ward (day 19); discharge (day 36)

Definition of abbreviations:

R = Right; L = Left; CTPA, computed tomography pulmonary angiography; PE, pulmonary embolism; PEEP, Positive End-Expiratory Pressure; RUL, right upper lobe; RVSP, right ventricular systolic pressure; PH, pulmonary hypertension; UFH, unfractionated heparin; POD, postoperative day; LUL, left upper lobe; PA, pulmonary artery; LPA, left pulmonary artery; LMWH, low molecular weight heparin; RV, right ventricle; IVC, inferior vena cava; CTEPH, chronic thromboembolic pulmonary hypertension; PTE, pulmonary thromboembolism; CXR, Chest X-Ray; OSA, obstructive sleep apnea; IJ, internal jugular vein; OAC, oral anticoagulant; PEA, pulmonary endarterectomy; RPA, right pulmonary artery; ER, emergency room; VV-ECMO, Veno-Venous Extracorporeal Membrane Oxygenation.

Overall, current evidence from case reports and small-scale studies demonstrates that EIT can identify regional perfusion defects and V/Q mismatch in patients with PE, even when ventilation remains preserved. In critically ill or postoperative patients, EIT has provided valuable bedside information when conventional imaging was delayed or contraindicated. Moreover, EIT also shows potential for dynamic monitoring of therapeutic effects. For instance, after adjustment of anticoagulation, thrombolysis, or ventilatory parameters, the perfusion signal gradually improves, which is consistent with the imaging of embolus absorption.

### Imaging features and manifestations of perfusion defects

Based on the above case reports and small clinical studies, a characteristic finding of acute PE on EIT is a focal, segmental pattern of ventilation–perfusion mismatch. Specifically, in EIT perfusion imaging, PE is often manifested as markedly weakened or absent perfusion signals in one lung or specific regions, while ventilation images remain relatively preserved, resulting in a characteristic V/Q mismatch. It should be emphasized that the V/Q mismatch identified by EIT reflects a spatial dissociation between ventilation and perfusion rather than an alteration in physiological V/Q ratios. Similar to conventional perfusion imaging, EIT shows significantly decreased impedance variation in perfusion-deficient areas, with well-defined regional boundaries, which can be valuable for bedside dynamic monitoring of disease progression or treatment response. Multiple animal studies have confirmed that hypertonic saline–enhanced EIT perfusion imaging correlates well with CTPA or SPECT perfusion imaging (Frerichs et al., 2002; Borges et al., 1985; Hentze et al., 2018). Preliminary clinical studies and case reports have also demonstrated good agreement between EIT perfusion imaging and CTPA findings (He et al., 2020b; Safaee et al., 2020; He et al., 2020c; Yuan et al., 2021).

**FIGURE 1:**
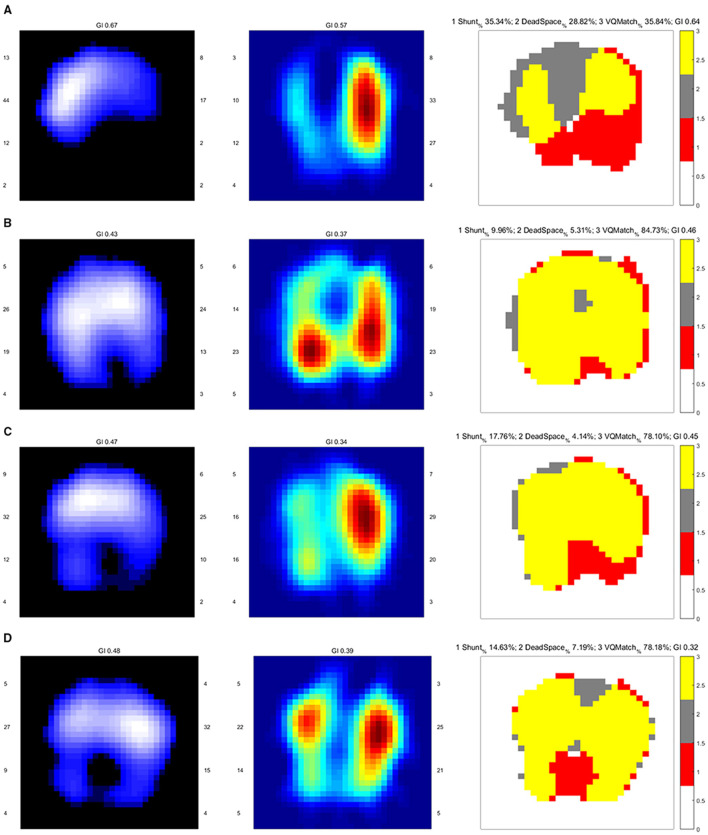
Typical EIT perfusion imaging patterns in different types of pulmonary embolism (Wang et al., 2021). **(A)** Marked ventilation defect in the dorsal lung and a significant perfusion defect in the right lung. **(B)** Partial restoration of right lung perfusion following thrombolytic therapy. **(C)** Ventilation defect in the left dorsal lung with pronounced shunt visible on the ventilation/perfusion (V/Q) ratio distribution image. **(D)** Ventilation defect in the dorsal lung with symmetric perfusion observed in both lungs.

In addition, several reports have shown that perfusion defects observed on EIT images gradually improved following thrombolytic or anticoagulant therapy, suggesting its potential utility in treatment response evaluation (Yuan et al., 2021; Wang et al., 2021; Prins et al., 2023; Ding et al., 2024). The figure below illustrates the typical manifestations of EIT perfusion imaging in different types of PE (Figure 1) (Wang et al., 2021).

### Cross-study comparison of EIT diagnostic parameters in clinical research

By reviewing relevant literature, we have initially collated the main diagnostic parameters and calculation methods of EIT currently used for the assessment of PE (Table 3).

**TABLE 3:** Major diagnostic parameters and calculation methods for EIT in the evaluation of pulmonary embolism.

Diagnostic parameter	Definition	Calculation method
Perfusion Distribution Inhomogeneity	Markedly lower perfusion signals in suspected embolic regions compared to normal areas	Affected side perfusion percentage = (ΔZ of affected side/Sum of bilateral ΔZ) × 100% (Borges et al., 1985)
V/Q mismatch	Focal mismatch between ventilation (normal) and perfusion (reduced)	Assess concordance between ventilation and perfusion signals within the same ROI.
V/Q correlation coefficient	Correlation between ventilation and perfusion signals over time in a selected ROI.	Lower correlation indicates greater V/Q mismatch
Impedance-derived V/Q index	Ratio of ventilation amplitude to perfusion amplitude in a region	ΔZ_V_/ΔZ_Q_ (abnormally elevated in the perfusion defect area.)
Dead Space–Related Indices	Proportion of lung with ventilation but no perfusion	$D\text{ead space}\%=R_{v}/\left(R_{v}+R_{p}+R_{v+p}\right)\times 100\%$
Defect area percentage	Percentage of mismatched area relative to total or ipsilateral lung field	Apply threshold to V/Q classification maps; calculate area ratio

Definition of abbreviations:

ΔZ, impedance change amplitude synchronous with heartbeat (perfusion) or respiration (ventilation).

ΔZ_V_, synchronous with respiration; ΔZ_Q_, synchronous with cardiac pulsation.

ROI, region of interest; typically divided into 4 quadrants or left/right halves. (Scaramuzzo et al., 2024).

(On this basis, to further simplify bilateral comparison, EIT, images may also be divided along the median line into the left and right ROIs.).

R_V_, Ventilation-only region; R_P_, Perfusion-only region; R_V + P_ = Ventilation–perfusion matched region.

However, these parameters have not been widely adopted in clinical practice, primarily due to significant technical and methodological heterogeneity, which has made it difficult to establish uniform diagnostic thresholds. Firstly, the processing workflow of EIT involves multiple critical steps, such as image reconstruction, filtering parameter settings, and ECG gating strategies. However, there is currently a lack of internationally standardized operating protocols for these steps, leading to variations in the fundamental characteristics of images generated by different devices or research teams. Secondly, differences also exist in the definition of the region of ROI and its reference baseline. Whether using geometric division based on the entire image or physiological division based on functional lung contours, the specific implementation involves a certain degree of subjectivity or algorithm dependency. This reduces comparability across different studies and affects the reproducibility of research findings. Furthermore, diversity exists in the core algorithms and parameter definitions themselves, such as the separation of ventilation and perfusion signals, calculation of V/Q correlation, and models for estimating dead space fraction. The use of different calculation methods in various experimental studies makes data from different sources difficult to compare or integrate directly.

**FIGURE 2:**
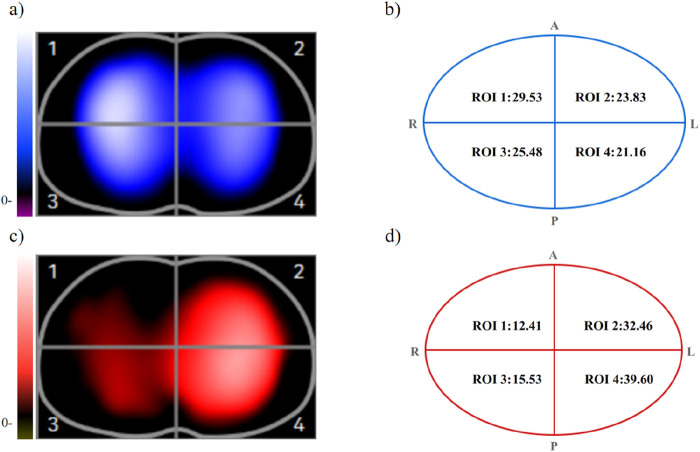
Pulmonary ventilation and perfusion imaging. **(a)** Ventilation distribution (blue); **(b)** Relative ventilation contribution of each quadrant, evenly distributed; **(c)** Perfusion distribution (red); **(d)** Relative perfusion of each quadrant (insufficient perfusion in the upper lobe of the left lung). Source: Unpublished data provided by Dr. Huiting Li, Department of Pulmonary Circulation, Shanghai Pulmonary Hospital, Tongji University (Infivision ET1000). Used with permission.

**FIGURE 3:**
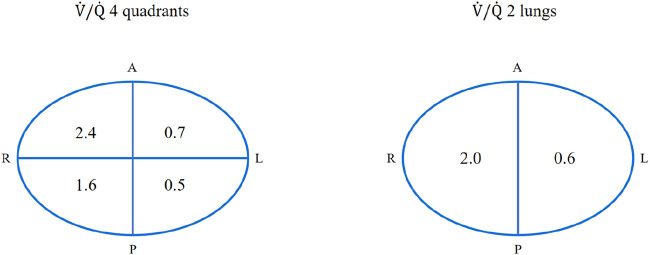
Ventilation/Perfusion Ratio in Each Quadrant. The left panel displays quadrant-specific V/Q ratios (values: 2.4, 1.6, 0.7, 0.5), revealing substantial intra-pulmonary heterogeneity. The right panel summarizes the integrated V/Q ratios for the entire right (2.0) and left (0.6) lungs, demonstrating a pronounced lateral imbalance. Source: Unpublished data provided by Dr. Huiting Li, Department of Pulmonary Circulation, Shanghai Pulmonary Hospital, Tongji University (Infivision ET1000). Used with permission.

In order to make the textual description more concrete, we have included representative figures (Figures 2, 3), derived from unpublished data courtesy of Dr. Huiting Li (Department of Pulmonary Circulation, Shanghai Pulmonary Hospital, Tongji University; Infivision ET1000) and used with permission, hoping that readers can gain a clear understanding of EIT imaging and segmentation.

This review further compares the numerical values of EIT-derived parameters reported across different clinical studies (Table 4). Notably, substantial variability exists in the absolute values of the same diagnostic parameter among studies. For example, the reported dead space% in patients with PE ranges broadly from approximately 30%–50%, and similar variability is observed in left–right perfusion ratios and V/Q mismatch proportions. Such inter-study discrepancies primarily stem from methodological heterogeneity—including differences in contrast agent concentration, breath-holding strategies, ROI segmentation, image reconstruction algorithms, and analytical thresholds. These sources of variation explain why a universal quantitative diagnostic cutoff for EIT has not yet been established. Nevertheless, these parameters retain significant value for trend monitoring and dynamic bedside assessment.

**TABLE 4:** Comparison of EIT diagnostic parameters used for pulmonary embolism assessment across clinical studies.

Parameter	Study	Reported values (PE group/Control group)	Methodological features	Key findings	Sources of variability (inter-study differences)
Dead space %	He H. et al. (2021) (n = 11)	PE: Significantly elevated (43.1% ± 11.1%) Non-PE: 5.7% ± 9.2%	10 mL 10% hypertonic saline; breath-hold; ROI segmentation	Elevated dead space fraction may indicate PE.	Contrast agent concentration, breath-hold duration, ROI segmentation method
	Safaee et al. (2020) (n = 1)	Dead space = 66% (Pre-treatment)	10 mL 10% hypertonic saline; breath-hold; ROI segmentation	Dead space decreased following treatment, demonstrating potential for dynamic monitoring	Single-case study; results may be highly variable
	Wang et al. (2021) (n = 1)	Dead space = 28.2% (pre-thrombolysis) → decreased (post-thrombolysis)	10 mL 10% hypertonic saline; breath-hold; ROI segmentation	Dead space improvement correlates with thrombolytic efficacy	No threshold could be defined
Perfusion ratio	Safaee et al. (2020) (n = 1)	L/R = 64%/36% (PE) → improved post-treatment	10 mL 10% hypertonic saline; breath-hold; ROI segmentation	Uneven infusion is an important signal for PE.	Differences in ROI segmentation, measurement techniques, and image reconstruction algorithms
V/Q mismatch area%	He et al. (2020b) (n = 11)	PE group: V/Q matched area significantly decreased; mismatched area significantly increased	10 mL 10% hypertonic saline; functional segmentation; threshold = 20% of maximum	V/Q mismatch is the most stable characteristic of PE.	Threshold definition differs from other studies
	Yuan et al. (2021) (n = 1)	Bilateral V/Q mismatch; improved post-thrombolysis	10 mL 10% hypertonic saline; breath-hold; ROI segmentation	Quantifiable therapeutic response	The single-case study lacks comparability
Perfusion defect area %	Ding et al. (2024) (n = 1)	Right lung perfusion was markedly reduced; improved but remained asymmetric on day 28	10 mL 10% hypertonic saline; ROI segmented by lung side	The defect can persist, reflecting a chronic/subacute condition	Differences in ROI segmentation method
Regional V/Q ratio	Grassi et al. (2020) (n = 1)	V/Q = 22%/7% in the left upper lobe (PE region)	Intravenous injection of a small amount of normal saline	Abnormal V/Q ratio suggests focal PE.	Lack of standardization in contrast agent
RV, RP %	He et al. (2020b) (n = 11)	PE group: RP (region of perfusion deficit) was significantly increased Non-PE group: RP accounted for a low proportion	Tri-zone segmentation method	RP region is expanded in PE patients	Threshold for zone segmentation is highly subjective

### Compared with traditional imaging techniques

Although EIT cannot yet replace standard imaging modalities such as CTPA, it offers several unique advantages: it does not require patient transfer, making it particularly suitable for critically ill patients in the ICU; it provides strong dynamic monitoring capability, enabling multi-timepoint evaluation of perfusion changes and real-time observation during thrombolysis; it allows integration with ventilation images to facilitate identification of V/Q mismatch; and it is radiation-free and contrast-free, making it applicable to patients with contraindications.

Nevertheless, EIT still faces several limitations. According to GRADE evidence-based criteria, the current level of clinical evidence supporting the use of EIT for PE diagnosis remains low. Most available studies consist of case reports and small sample analyses, with only one prospective study among the 14 studies summarized. In addition, substantial methodological heterogeneity exists across studies, including variability in hypertonic saline concentrations and differences in image-processing algorithms, making it difficult to draw robust conclusions. Furthermore, the limited spatial resolution of EIT constrains its ability to detect subsegmental or more distal pulmonary emboli, and its diagnostic sensitivity remains markedly inferior to that of CTPA (He et al., 2020b). Most studies also failed to report the time interval between EIT and CTPA examinations, limiting the ability to evaluate the true timeliness of EIT as an early screening tool.

### Innovative development

At present, EIT has been preliminarily applied in the clinical management of PE. Overall, its role in diagnosis appears relatively more established, while its use in therapeutic monitoring remains limited, for example, comparing perfusion before and after thrombolysis. Furthermore, its diagnostic sensitivity is still inferior to that of CTPA (She et al., 2023). An ideal clinical strategy would be to integrate these modalities based on patient conditions: using EIT for early bedside imaging and continuous monitoring, confirming the diagnosis with CTPA in stable situations, and employing echocardiography for cardiac function assessment, thereby enabling a more precise and safer individualized treatment plan.

Based on the above case studies, we further refined this integrated pathway. For ICU patients with high-risk factors or clinical instability, EIT should first be utilized for bedside monitoring. When unexplained desaturation or hemodynamic fluctuations occur, an immediate EIT examination is warranted. If the images demonstrate a clear regional V/Q mismatch, this can serve as an important basis for initiating empirical anticoagulation, and such patients may be classified as “highly suspicious.” For patients with positive EIT findings and relatively stable vital signs, confirmatory imaging and risk stratification should be performed using conventional modalities. Simultaneously, bedside echocardiography should be used to assess right ventricular function and identify acute cor pulmonale, thereby providing hemodynamic evidence to guide subsequent therapeutic decisions.

Once treatment has begun, the utility of EIT can be extended further. EIT is capable of continuously tracking dynamic changes in perfusion defects, allowing clinicians to objectively assess the actual response to anticoagulation or thrombolytic therapy and to adjust treatment strategies in a timely manner. Of course, the clinical effectiveness and workflow feasibility of this integrated approach require validation through future prospective studies.

## Challenges in clinical translation and future directions

### Motion artifacts and advances in three-dimensional imaging

Current mainstream two-dimensional EIT systems are susceptible to artifacts caused by physiological activity occurring outside the imaging plane, such as diaphragmatic motion or changes in body position. These artifacts may compromise image quality and reduce the accuracy of regional localization. The development of three-dimensional EIT offers a promising solution to this challenge. By deploying dual electrode belts for simultaneous data acquisition and applying three-dimensional reconstruction algorithms, full-volume lung imaging can be achieved. This approach not only helps reduce motion-induced artifacts but also improves the anatomical accuracy of lesion localization (Grychtol et al., 2019; Gao et al., 2024). Efforts to optimize signal quality are also ongoing. Hyun et al. developed a novel automated signal quality index (SQI) method using discriminant models and manifold learning to detect abnormal CVS induced by motion artifacts, representing the first attempt to enhance EIT cardiopulmonary monitoring by assessing CVS signal quality (Min Hyun et al., 2023).

### Challenges of spatial resolution and computational complexity, and the empowering role of AI

EIT is inherently limited by its low spatial resolution and modest conductivity contrast, which can make the resulting images difficult to interpret. Achieving high-quality three-dimensional imaging places additional demands on hardware, requiring more sophisticated electrode arrays and substantially more complex reconstruction algorithms. To overcome these bottlenecks, researchers have increasingly integrated advanced computational methods into EIT reconstruction.

Dong Liu et al. applied convolutional neural network (CNN)-induced regularization with deep image prior (DIP) to EIT reconstruction, offering a novel approach to regularization in EIT inverse problems (Liu et al., 2023). Similarly, Junwu Wang et al. proposed using image priors to guide neural network initialization, thereby improving EIT image reconstruction quality (Wang et al., 2025). Together, these studies highlight the impact of integrating modern computational techniques and neural network architectures on advancing EIT technology, providing more accurate, efficient, and versatile imaging solutions for medical and scientific applications.

Collectively, these studies illustrate the impact of integrating advanced computational methods and neural network architectures on advancing EIT technology, providing more accurate, efficient, and versatile imaging solutions for medical and scientific applications.

### Ethical considerations and practical recommendations for clinical integration

Given the current technological limitations of EIT, its clinical application should adhere to prudent ethical standards. EIT should be regarded as part of an integrated diagnostic pathway rather than an independent diagnostic tool. For patients in whom EIT screening suggests a high suspicion of PE or presents complex findings, confirmation with higher-precision imaging modalities such as CTPA is essential. Moreover, prior to clinical use, patients and their families should be adequately informed of the benefits, risks, and limitations of the technique.

With continuous improvements in digital image quality and data processing algorithms, the technical bottlenecks of EIT in pulmonary perfusion imaging are gradually being overcome. Advances in EIT technologies, including the integration of AI and novel sensors, are opening a new era of EIT research.

## Summary and outlook

After decades of development, EIT has evolved into a theoretically mature imaging technique with broad clinical prospects. Its noninvasive, real-time, and continuous monitoring capabilities of ventilation and perfusion distribution provide a new tool for the diagnosis and management of respiratory diseases. Both clinical and experimental studies have confirmed good consistency between hypertonic saline–based EIT perfusion imaging and conventional modalities such as CTPA or SPECT. In addition, multiple case reports have demonstrated that perfusion defects observed on EIT gradually recovered following thrombolytic or anticoagulant therapy, suggesting potential utility in treatment response monitoring. By capturing the characteristic V/Q mismatch of PE, EIT is regarded as a promising bedside diagnostic tool, particularly for critically ill patients who cannot undergo immediate CTPA, thus providing timely support for clinical decision-making.

Nevertheless, EIT for pulmonary perfusion imaging remains in the early stages of clinical translation. Key limitations include insufficient spatial resolution, susceptibility of image reconstruction to artifacts and nonlinear inverse problems, and the absence of standardized clinical protocols. Looking ahead, advances in three-dimensional reconstruction algorithms, integration of artificial intelligence, and validation through combination with established imaging modalities such as CTPA are expected to help overcome current barriers and further expand the role of EIT in bedside detection and dynamic monitoring of PE.
