# Correction: Bedside detection and monitoring of pulmonary embolism using electrical impedance tomography

**DOI:** 10.3389/fphys.2026.1800500

**Published:** 2026-02-18

**Authors:** Mingyuan Deng, Nianze Li, Jiafeng Wang, Shuang Zhao, Mingjing Yu

**Affiliations:** 1 West China School of Medicine/West China Hospital, Sichuan University, Chengdu, China; 2 Department of Respiratory and Critical Care Medicine, Institute of Respiratory Health, State Key Laboratory of Respiratory Health and Multimorbidity, West China Hospital, Sichuan University, Chengdu, China

**Keywords:** bedside monitoring, critical care, electrical impedance tomography (EIT), pulmonary embolism (PE), pulmonary perfusion

The funder “Science and Technology Department of Sichuan Province-International Science and Technology Innovation Cooperation Project,” grant number “2024YFHZ0273” to Mingjing Yu was erroneously omitted. Additionally, the funder “1·3·5 Project of State Key Laboratory of Respiratory Health and Multimorbidity, West China Hospital, Sichuan University,” grant number “RHM25213” to Shuang Zhao was erroneously omitted. The correct funding statement reads:

“The author(s) declared that financial support was received for this work and/or its publication. This work was supported by the Zhong Nanshan Medical Foundation of Guangdong Province (ZNSXS-20240095), Science and Technology Department of Sichuan Province-International Science and Technology Innovation Cooperation Project (2024YFHZ0273) to author MY, and 1·3·5 Project of State Key Laboratory of Respiratory Health and Multimorbidity, West China Hospital, Sichuan University (RHM25213) to author SZ.”

The original article has been updated.

